# Comparison of Five Different Treatment Approaches of Mandibular Keratocystic Odontogenic Keratocyst (OKC): A Retrospective Recurrence Analysis of Clinical and Radiographic Parameters

**DOI:** 10.1007/s12663-023-01929-0

**Published:** 2023-06-21

**Authors:** Henriette L. Moellmann, Aida Parviz, Marcia Goldmann-Kirn, Madiha Rana, Majeed Rana

**Affiliations:** 1 University Hospital Duesseldorf, 40225 Duesseldorf, Germany; 2 Department for Craniomaxillofacial Surgery, Hannover Medical School, Hannover, Germany; 3 Department of Psychology, University of Applied Sciences, 22143 Hamburg, Germany

**Keywords:** Keratocystic odontogenic tumor/odontogenic keratocyst, Odontogenic tumor, Multicystic intraosseous tumor, Virtual surgical planning (VSP), Computer-assisted surgery (CAS)

## Abstract

The odontogenic keratocyst (OKC) is a benign but locally aggressive growing lesion that infiltrates the bone and surrounding tissue. It is characterized by high rates of recurrence along with rapid growth. Different forms of partly successful treatment therapies are reported. The retrospective study at hand examined 114 patients with OKC treated over a period of 20 years. Data extracted includes gender, age, location, previous treatment for the lesion, surgery, outcome, recurrence rate and follow-up. 63.1% of the patients underwent cystectomy, 22.5% by cystectomy and carnoy solution, 7.2% by cystectomy, and curettage, 4.5% by cystostomy and 2.7% by partial resection. In this study, no significant differences could be observed regarding the surgical method. Most recurrences occurred with 91.9% in the mandible with an average size of 5.5 cm^2^ and increased in women. Within a mean follow-up time of 3.6 years the recurrence rate was 36.9%, on average after 36 months. Recurrences were most frequently diagnosed at the age of 31–50 (43.9%). Despite numerous studies, there is still no unanimous opinion on an effective therapy for OKC. However, precise resection of OKC can be facilitated by preoperative 3D-imaging and virtual planning.

## Introduction

The World Health Organization (WHO) classifies the odontogenic keratocyst (OKC) as a developmental (disembriogenetic) cyst in 1992. In 2005 the WHO categorized the lesion as an ondontogenic tumor (keratocystic odontogenic tumor, KCOT) [1]. The novel term expresses its neoplastic nature as “a benign uni- or multicystic, intraosseous tumor of odontogenic origin, with a characteristic lining of parakeratinized stratified squamous epithelium and potential for aggressive, infiltrative behavior”. The WHO pusblished the 4th edition of the ‘Classification of Head and Neck Tumors’ in January 2017, in which the KCOT is again classified as an OKC [2]. The KCOT or OKC is a benign uni- or multicystic, intraosseous tumor. It is of odontogenic origin and shows a characteristic aggressive, infiltrative behaviour [1, 3]. The OKC is considered an odontogenic cyst first reported by Philipsen in 1956 and attracted interest because of its pathological features as well as its high recurrence rate [4]. KCOT or OKC occur over a wide age range [5] and it is more frequent amongst men than women [6]. Numerous studies have concluded that the mandible is perpetually infiltrated compared to the maxilla, particularly the posterior body and ascending ramus [6]. Clinical symptoms include pain, swelling and hypaesthesia. However, there are patients without any clinical symptoms that are diagnosed accidentally or in late stages, e.g., developing pathological fractures [6]. There are reports on OKC penetrating the surrounding soft tissues [7], base of skull [8], orbit and infratemporal fossa [9]. In radiological images, OKC appears as a unilocular or multilocular radiolucency with an irregular contour [10]. Histologic features include a thin layer of epithelium, a basal cell layer consisting of palisading cuboidal or columnar cells, and a luminal surface. Furthermore, the OKC shows satellite cells in connective tissue [11]. Being one of the major diagnosis criteria, OKC recurrently occurs in association with basal cell nevus syndrome/Gorlin Goltz syndrome. This is an autosomal dominant syndrome characterized by basal cell carcinomas, intracranial calcifications (especially of falx cerebri), and distinct facial abnormalities [12].

Surgical therapy of OKC is still controversial [13]. On the one hand, there are conservative therapies including an enucleation (with or without curettage), a marsupialization, or decompression [14]. On the other hand, there are ostectomy (curettage with Carnoy’s solution, cryotherapy or electrocautery) or the resection. The choice of the treatment depends on the size of the cyst, the location and patients’ age [15]. When choosing the appropriate therapy, reduction of the recurrence rate and the minimization of morbidity must be considered [14].

Despite numerous studies, there is still no consistent opinion on the effective therapeutic treatment of KCOT.

The aim of the present retrospective study was to record and analyse the clinical behaviour of KCOT as well as its occurrence of recurrences to be able to make treatment recommendations and to place these in the overall context of the scientific literature.

## Material and Methods

This retrospective study is based on 114 patients with OKC who were operated from 1990 to 2010 and was approved by the local ethics committee. Medical records were analyzed by using a self-developed data registration form. The following parameters were queried: age, age at initial diagnosis, gender, medical history, symptoms, location, Relationship to surrounding tissues (teeth, n. alveolaris inferior), radiological expansion, previous treatment for the lesion, surgery, time until first recurrence rate and follow-up. The collected data were recorded with Excel and processed using the SPSS 21.0 (IBM Corp. Released 2012. IBM SPSS Statistics for Windows, Version 21.0. Armonk, NY: IBM Corp) statistics program. The explorative data analysis was used for the descriptive evaluation and for the examination of special peculiarities of the data.* T*-test was used to check whether two independent variables (e.g., age/recurrence) also had two mean values of different magnitudes in the population and not only in the case group. This allowed the comparison an interpretation of the significance between the occurrence of a relapse and a particular variable in the population. To record correlations between two variables (e.g., recurrence/gender) in our collective, we used a cross table and tested with the Chi-square test whether these correlations can be transferred to the whole. We further conducted the Kaplan–Meier analysis to assess time to recurrence regarding the different surgical methods, gender, age, and significance.

## Results

Three out of 114 patients (2.6%) suffered from NBCCS. In the investigated collective, men were affected twice as often (66.7%; 74/111) as women (33.3%; *n* = 37/111). At initial diagnosis, men were on average 44 and women 41 years old. The tumor most frequently occurs in the age group of 31–50-year-olds (37.8%; *n* = 42/111). 91.9% of the OKC were found in the mandible commonly in the angle between the jaw and the mandibular branch (64.0%). Only 8.1% were located in the maxilla. On average the cysts had a size of 5.5 cm^2^ (measured mesio-distal, cranio-caudal).

The following methods were used in the investigated collective of patients (see Table 1):

**Table 1:** Overview surgical methods

Surgical method	Number of patients (*n*)	Percentage (in %)
CO	25	4.5
CE	70	63.1
CE + CS	5	22.4
CO + CU	8	7.2
PRES	3	2.7
CRES	0	0

*CO* Cystostomy;* CE * Cystectomy;* CE + CS * Cystectomy and Carnoy solution;* CO + CU * Cystectomy and curettage;* PRES* Partial resection of the jaw;* CRES* Continuity resection

In most cases (*n* = 62, 55.9%), the defect after surgery needed no augmentation. In 44.1%, an augmentation was necessary (see Table 2).

**Table 2:** Overview method of augmentation

		Method of augmentation						Total
		No treatment	Collagen-vlies	Osteo-synthesis with bone via bone-scraper	Osteo-synthesis with cancellous bone from iliac crest	Osteo-synthesis with mono-, bi-cortical bone	Osteo-synthesis + collagen-vlies	
	CO	15	0	0	10	0	0	25
	CE	36	1	1	26	4	2	70
	CE + CS	5	0	0	0	0	0	5
	CO + CU	4	1	0	1	2	0	8
	PRES	2	0	0	1	0	0	3
	CRES	0	0	0	0	0	0	0
Total		62	2	1	38	6	2	111

*CO* Cystostomy;* CE * Cystectomy;* CE + CS * Cystectomy and Carnoy solution;* CO + CU * Cystectomy and curettage;* PRES* Partial resection of the jaw;* CRES* Continuity resection

Postoperative monitoring of the patient population was based on the clinical examination and radiological findings over a period of 2 months to 17 years (202 months). The mean value of the follow-up times was 41.9 months (3.5 years), the median value 24 months (2.0 years). 70 patients (63.1%) remained without recurrence within the study period of 20 years. 41 patients had relapses during this period. The recurrence rate was 36.9%.

**Fig. 1:**
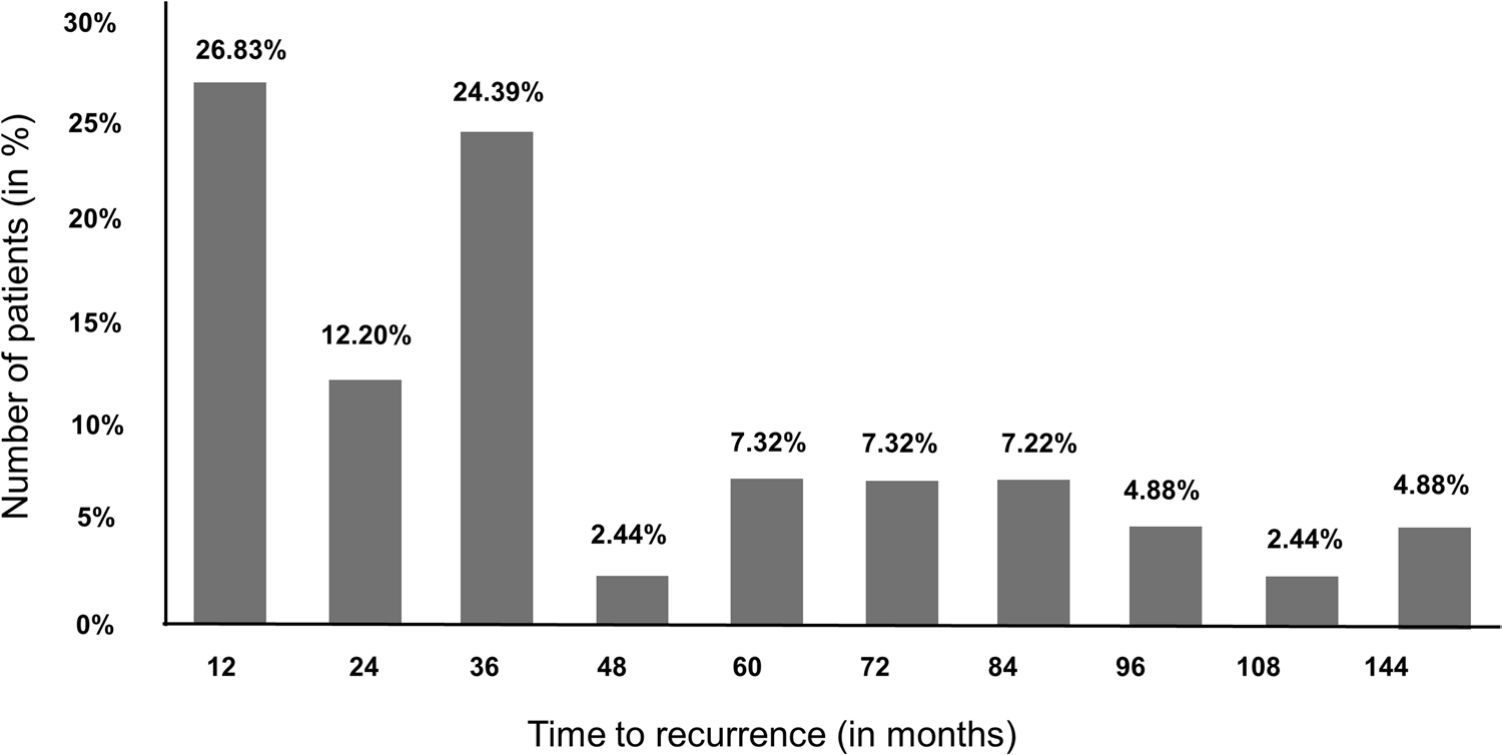
Frequency of occurrence of the 41 recurrences by month (in percent)

In 27 (65.8%) of the 41 patients with recurrence, recurrence occurred in the first four postoperative years with a decreasing tendency. From the fifth postoperative year onwards, 14 patients (34.1%) were still suffering from recurrence. On average, recurrences occurred after approximately 36 months (mean = 46.2 months, see Fig. 1).

Regarding localization, most recurrences occurred unilaterally in the fourth quadrant (*n* = 23) or in the third quadrant (*n* = 16). Only two recurrences were found in the upper jaw. The recurrence in the upper jaw differed in time from the recurrence in the lower jaw. In the maxilla the median recurrence occurred after 18 months (1.5 years), in the mandible after 36 months (3 years) postoperatively, so that the difference was 18 months (1.5 years).

**Fig. 2:**
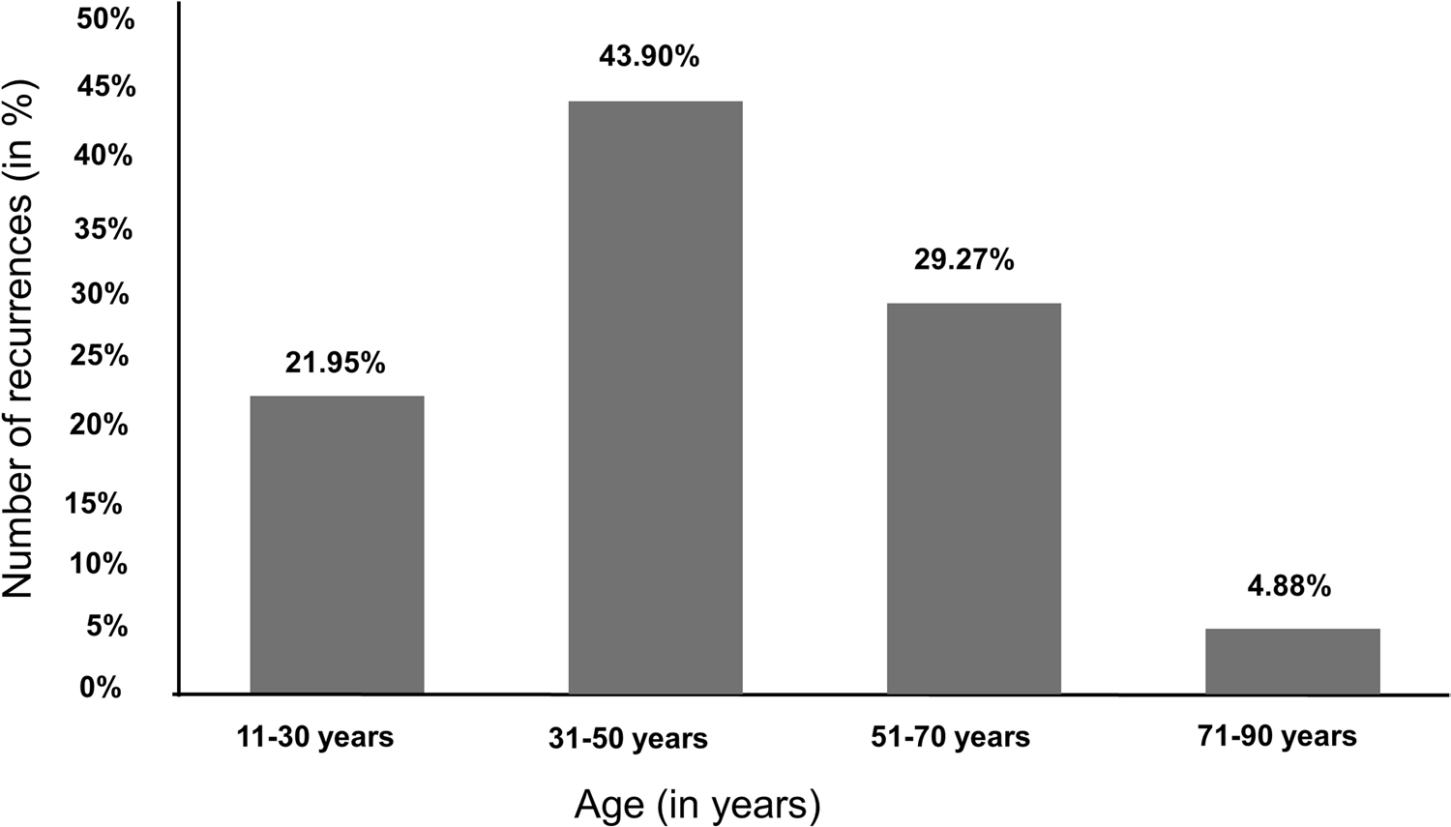
Frequency distribution of recurrences within age groups (in percent)

The average age at recurrence was 44 years. The group of 31–50-year-olds with 43.9% was most frequently affected by recurrences, followed by the age group of 51–70-year-olds (29.2%, see Fig. 2).

**Fig. 3:**
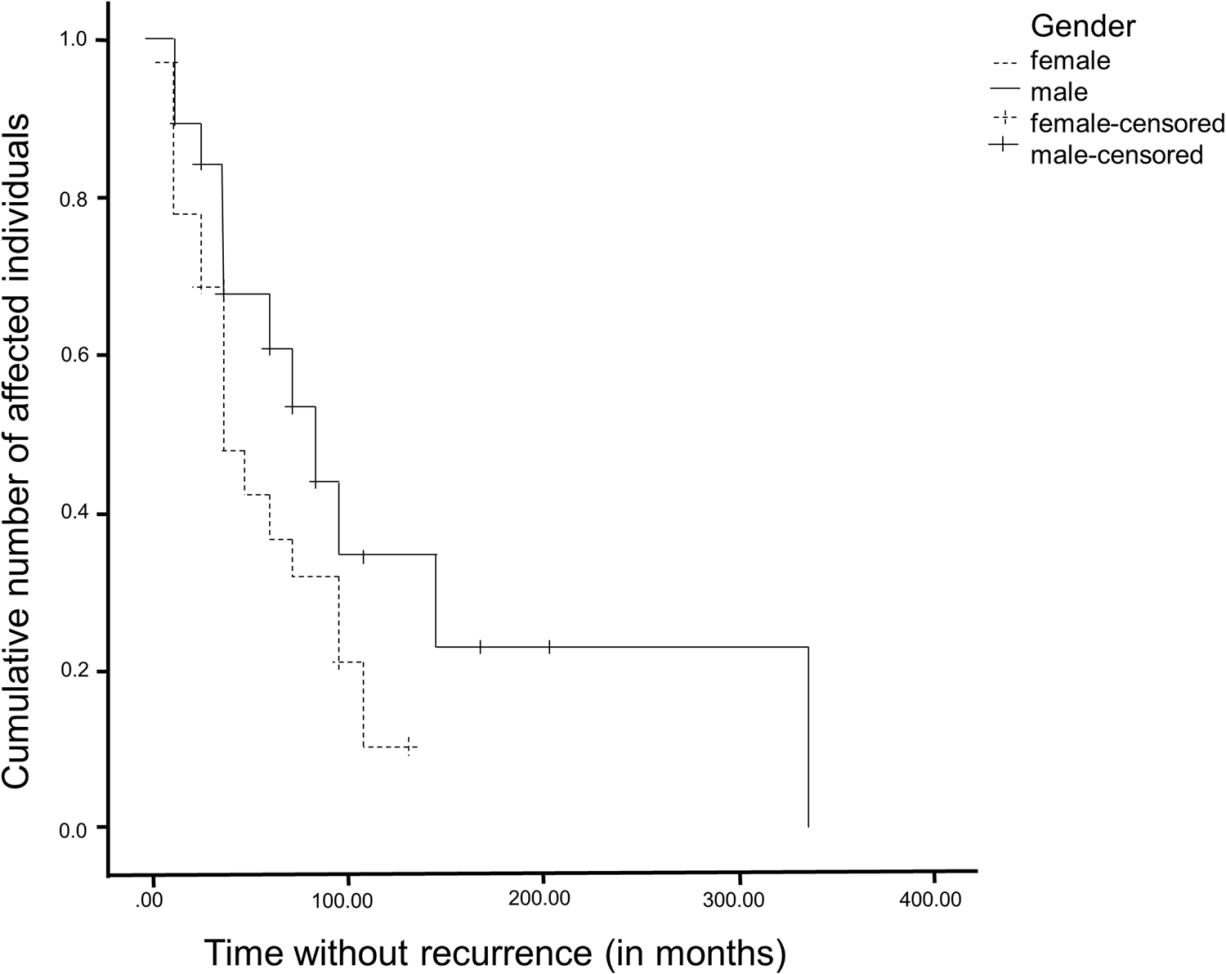
Recurrence-free time in months

**Fig. 4:**
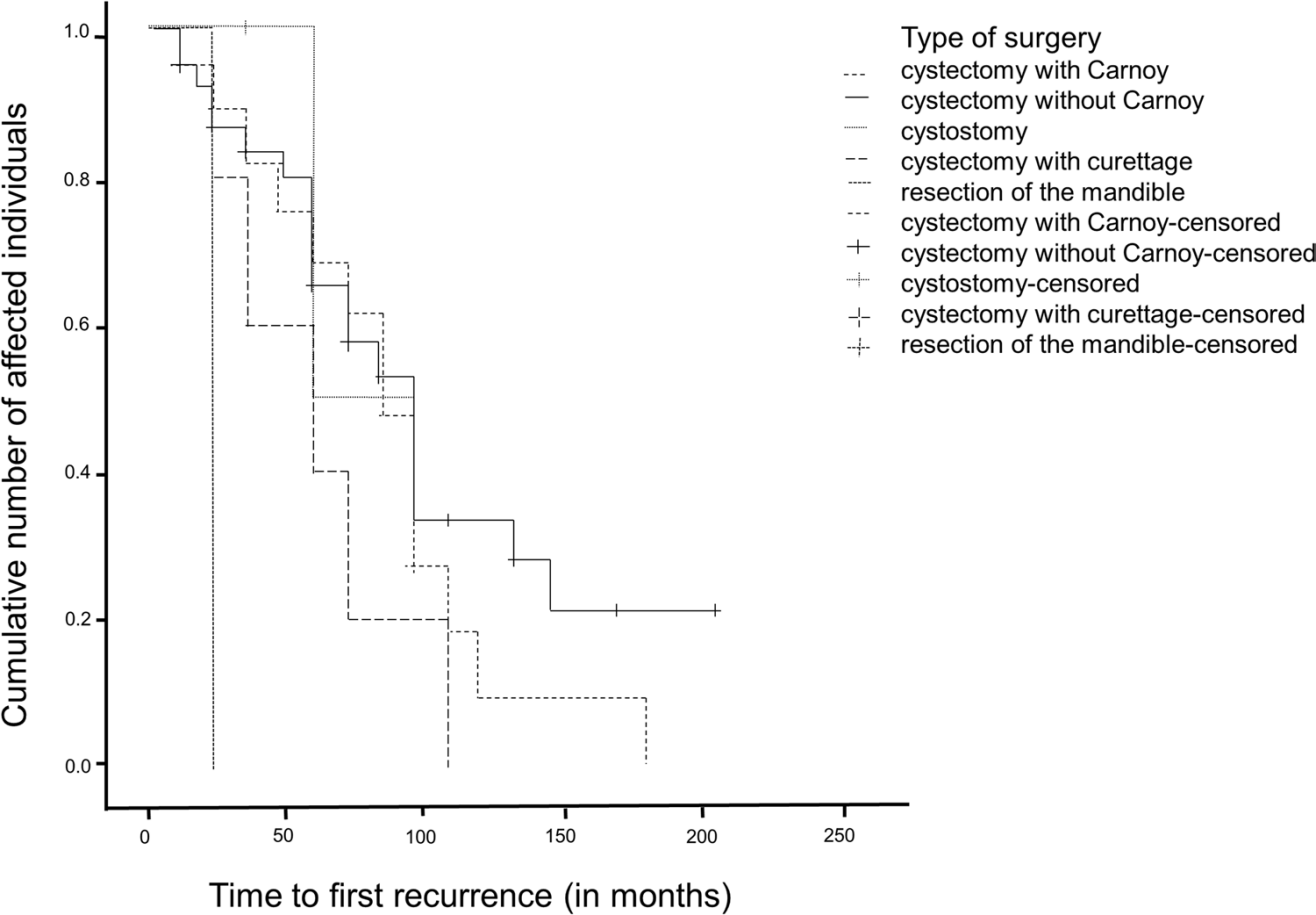
Observation time until first recurrence (in months)

According to the Kaplan–Meier analysis, recurrences occurred after 60 months in 50% of the 31–50-year-old patients, and after 84 months (51–70-year-old). A significance between the time of recurrence after the first operation and a certain age group could not be demonstrated (*p* = 0.869). Within the total number of 41 recurrences, 18 women (43.9%) and 23 men (56.1%) suffered from recurrence. On a gender-specific basis, 48.6% of women (18/37) and 31.1% of men (23/74) received a recurrence within the study period. Using the Chi-square test, no statistically significant relationship could be established between the occurrence of a recurrence and the gender (*p* = 0.071). The median relapse-free, postoperative time, after which 50% of the women received a recurrence, was 36 months (3 years) within the study period. In comparison, 50% of men received a recurrence after 84 months (7 years). These values show a time difference of 48 months (4 years) between the recurrence of the tumor and the gender. The Kaplan–Meier analysis showed a *p*-value of 0.053, which does not statistically support the differences (see Figs. 3 and 4).

Regarding the individual surgical methods, the following differences in recurrence rates were found: 28.6% after cystectomy, 56% after cystectomy with carnoy solution and 62.5% after cystectomy with curettage. The cystostomy showed 20% and the partial lower jaw resection showed a recurrence rate of 33.3%. Within the total number of *n* = 111 patients, the highest recurrence rate after cystectomy was observed (18%), followed by cystectomy with carnoy solution (12.6%) and cystectomy with curettage (4.5%). Partial resection and cystostomy showed the lowest recurrence rate with 0.9%. The chi-square test showed a* p*-value of 0.064 and thus did not provide a valid statement as to whether one surgical method is significantly better against the recurrence patient than another (see Table 3).

**Table 3:** Recurrence frequency depending on the surgical method

			Surgical method					Total
			CE + CS	CE	CO	CE with curettage	Partial resection of the jaw	
Recurrence	No	Number without recurrence	**11**	**50**	**4**	**3**	**2**	**70**
		% within no recurrence	15.7	71.4	5.7	4.3	2.9	100.0
		% within surgical method	44.0	71.4	80.0	37.5	66.7	63.1
		% of total number	9.9	45.0	3.6	2.7	1.8	63.1
	Yes	Number of recurrences	**14**	**20**	**1**	**5**	**1**	**41**
		% within no recurrence	34.1	48.8	2.4	12.2	2.4	100.0
		% within surgical method	56.0	28.6	20.0	62.5	33.3	36.9
		% of total number	12.6	18.0	0.9	4.5	0.9	36.9
Total		Total Number	**25**	**70**	**5**	**8**	**3**	**111**
		% within no recurrence	22.5	63.1	4.5	7.2	2.7	100.0
		% within surgical method	100.0	100.0	100.0	100,0	100.0	100.0
		% of total number	22.5	63.1	4.5	7.2	2.7	100.0

Bold values indicate the total number of recurrences

## Discussion

Only patients with confirmed histopathological findings and the diagnoses KCOT and OKC were included in the evaluation. All 114 patients underwent primary surgery between 1990 and 2010. Within this timespan, “keratocyst” were treated as a dysontogenetic cyst starting in 1991 and as a neoplasia of a tumorous process after 2005. The WHO’s classification, which in 2005 did justice to the new pathogenetic findings about the KCOT as a tumorous event, did not lead to any discernible change in treatment. It has also not been possible to reduce the enormous recurrence incidence of 2–62%, which is confirmed by the result of the present evaluation with a mean recurrence incidence of 36.9% [16]. Therefore, a better understanding of aetiopathogenesis and factors influencing relapse behavior as well as prognostically relevant factors to develop effective, low relapse therapies for patients seems crucial. With a median of 42 years (median women = 41 years old/median men = 44 years old), the age range extends from twelve to 90 years and is thus in agreement with a large part of the literature [17]. The large standard deviation of 19 years suggests that odontogenic keratocysts develop largely independently of age [18]. Men were twice as often affected by OKC as women in a ratio of 2:1. This is similar to the results described in the literature [19]. The OKC were found significantly more frequently (*p* < 0.001) in the lower jaw (91.9%) than in the upper jaw (8.1%). This conspicuous frequency and the predilection site in the posterior mandibular angle range with 68.3% are largely consistent with the current literature [17, 19–21]. The fact that the odontogenic keratocysts develop from development-related residues of Mallessez’s and Serres’ epithelial remains can explain the above-mentioned predilection in the mandible, since these cell residues often remain in the posterior region of the mandible during odontogenesis [22]. The vast majority of OKCs (59.5%) were found to be asymptomatic random findings in routine radiographic diagnostics [20]. The rather high proportion of asymptomatic odontogenic keratocysts in this study is due to a small radiological average size of 5.5 cm^2^ compared to the values of up to 19 cm^2^ given in the literature [23]. The connection between the size and the rather late symptoms in the lower jaw can be illustrated by the anterior–posterior growth direction in the cancellous bone. This allows the odontogenic keratocysts to assume considerable size before causing discomfort by perforating the compact bone [22]. The size data of other authors of up to 19 cm^2^ may explain their higher values of symptomatically diagnosed lesions [24]. Due to the predilection site in the toothless area of the posterior mandible, only 32.4% of the cases studied had a close spatial relationship to one or more teeth. 67.6% had no connection to teeth. In the literature, these data correlated with the size of the teeth and deviated from the values of the present study [10]. As in the literature, 98.2% of the KCOT were unilocular [20]. Large or multiple odontogenic keratocysts require an extended radiological diagnostic procedure such as a computer tomogram (CT) or a digital volume tomography (DVT) in order to determine the topographical-spatial extent more precisely and to be able to plan therapeutic measures more specifically [22]. In 45.9% of patients (*n* = 51), an augmentation procedure was required to restore functional stability of the jaw. Most frequently (*n* = 38) autogenous bone was removed from the iliac crest of the anterior superior iliac spine and used to fill the defect. This means an additional intervention for the extraction of bone material. However, clinical studies show that autogenous bone grafts remain the “gold standard’ in the augmentation technique. In the case of extensive bone defects, additional osteosynthesis plates may be required for stability in order to ensure prompt loading [25].

## Conclusion

Cystectomy showed the best results in terms of surgical method used and postoperative recurrence rate, but even this method could not reliably prevent recurrences. Nevertheless, many consider cystectomy alone to be an appropriate therapy because, in addition to being a gentle procedure, it can reduce the recurrence rate and prolong the recurrence-free period. Close follow-up is required for early detection of OKC. The follow-up intervals showed that annual clinical and radiographic control of the affected jaw section by three-dimensional imaging is recommended in the first three postoperative years. Basically, very long-term clinical and radiographic controls are recommended because recurrences can occur even 12 years after surgery. Further randomized, multicenter studies need to be performed to provide evidence-based treatment recommendations.

## Data Availability

The data presented in this study are available on request from the corresponding author. The data are not publicly available due to privacy regulations.
